# Downregulation of PTPRK Promotes Cell Proliferation and Metastasis of NSCLC by Enhancing STAT3 Activation

**DOI:** 10.1155/2019/4265040

**Published:** 2019-01-29

**Authors:** Xuting Xu, Dong Li, Jin Liu, Zhihong Ma, Huilian Huang, Lishan Min, Licheng Dai, Shunli Dong

**Affiliations:** ^1^Huzhou Key Laboratory of Molecular Medicine, Huzhou Central Hospital, Huzhou, Zhejiang 313000, China; ^2^Department of Thoracic Surgery, Huzhou Central Hospital, Huzhou, Zhejiang 313000, China; ^3^Department of Pathology, Huzhou Central Hospital, Huzhou, Zhejiang 313000, China

## Abstract

**Objective:**

The receptor-type tyrosine-protein phosphatase *κ* (PTPRK) is a candidate tumor suppressor involved in the tumorigenesis of various organs. However, its expression and biological roles in non-small-cell lung cancer (NSCLC) have not yet been investigated.

**Methods:**

PTPRK expression in NSCLC tissues and cell lines was examined using real-time PCR and western blotting. In addition, the effects of PTPRK on cell migration, invasion, and proliferation were evaluated *in vitro*. Furthermore, we explored whether the downregulation of PTPRK led to STAT3 activation in NSCLC cell lines by western blotting. The expression of phospho-STAT3^Tyr705^ in primary human NSCLC tissues was evaluated by immunohistochemistry.

**Results:**

The results showed that PTPRK expression was frequently reduced in NSCLC tissues with lymph node metastasis and cell lines. The inhibition of PTPRK expression resulted in increased proliferation, invasion, and migration of NSCLC cells *in vitro*. Additionally, after silencing of PTPRK, phospho-STAT3^Tyr705^ was significantly increased in NSCLC cells. Moreover, the phospho-STAT3^Tyr705^ levels of NSCLC tissues were positively correlated with lymph node metastasis and significantly inversely correlated with the expression of PTPRK (*p* < 0.05).

**Conclusions:**

These results suggested that PTPRK functions as a novel tumor suppressor in NSCLC, and its suppressive ability may be involved in STAT3 activation.

## 1. Introduction

Lung cancer, especially non-small-cell lung cancer (NSCLC), is the most frequent cause of cancer-related deaths [1]. Despite successful surgical resection and chemotherapy, the tumor recurrence happens frequently within 5 years with metastasis [2]. To date, the 5-year survival rate of lung cancer has not significantly increased [3]. In the past few decades, multiple oncogenes and tumor-suppressive genes have been discovered in the biological processes that regulate lung tumorigenesis. Nevertheless, the molecular mechanisms underlying pathogenesis are still poorly understood.

Alterations in tyrosine phosphorylation patterns are a common phenomenon in various human cancers, including lung cancer. Protein phosphorylation is a reversible process and is regulated by protein tyrosine kinases (PTKs) and protein tyrosine phosphatases (PTPs) [4, 5]. Receptor-type tyrosine-protein phosphatase *κ* (PTPRK), which resides in the frequently deleted chromosomal 6q region, is a transmembrane tyrosine phosphatase that contains an extracellular adhesion molecule-like domain and a cytoplasmic tyrosine phosphatase domain [6]. Recent studies have shown that PTPRK is frequently downregulated in many human cancers. For example, decreased PTPRK expression was reported in association with poor prognosis of breast cancer [7]. Other evidence suggested that PTPRK was a potential tumor suppressor in colon cancers [8]. Recent studies show that PTPRK is frequently underexpressed in NKTCL and contributes to NKTCL pathogenesis [9–11]. Although some studies have shown that the expression of PTPRK was significantly downregulated in lung cancer-derived cell lines, its contribution to aberrant signaling in lung cancers remains largely unexploited [12].

In the present study, we examined PTPRK expression in NSCLC tissues and cell lines and investigated PTPRK regulation in NSCLC progression.

## 2. Methods and Materials

### 2.1. Subjects and Clinical Data

Fresh tissue specimens were obtained from 46 patients who underwent surgical resection of NSCLC at the Huzhou Central Hospital from September 2013 to December 2015. None of the patients received any chemotherapy or radiation treatment prior to the surgery. The collected tissue samples were immediately frozen in liquid nitrogen and stored at -80°C before RNA isolation. Four *μ*m thick tissue sections used for immunohistochemistry were obtained from formalin-fixed paraffin-embedded tissue samples. Our study was approved by the ethics committees of the Huzhou Central Hospital. And written informed consent forms were acquired from all of the participants.

### 2.2. Cell Lines and Cell Cultures

Eight lung cell lines (16HEB, 95C, 95D, A549, GLC82, NCI-H1299, NCI-H460, and SPCA-1) were purchased from the Cell Bank of Chinese Academy of Medical Sciences (Beijing, China). The cells were cultured in a PRMI-1640 medium, supplemented with 10% fetal bovine serum (FBS) and 1.5 g/L sodium bicarbonate at 37°C in a humidified atmosphere of 5% CO_2_ (Thermo Electron Corp, USA).

### 2.3. RNA Extraction and Quantitative Real-Time PCR (qRT-PCR)

Total RNA was extracted from tissue specimens by using TRIzol™ Reagent (Thermo Fisher Scientific). The isolated RNA was converted to cDNA by the Prime-Script RT reagent kit (TaKaRa, Dalian, China). Quantitative real-time PCR analysis was conducted with the SYBR Premix Ex Taq TM II kit (TaKaRa) on an ABI 7500 Real-Time PCR System (Applied Biosystems, USA) and normalized to the expression of *β*-actin. The primers of PTPRK were forward 5′-ACAGAGTGGTGAAAATAGCAGGAA-3′ and reverse 5′-TGACAACTAGGAGAAGGAGGATGA-3′.

### 2.4. Immunohistochemistry

The formalin-fixed, paraffin-embedded tissue was sectioned (4 *μ*m) and mounted onto poly-L-lysine-coated glass slides for immunohistochemistry [13]. After being deparaffinized in xylene and rehydrated in a series of graded ethanol solutions, the slices were heated in a high-pressure cooker with 10 mmol/L of citrate buffer (pH 6.0) for antigen retrieval. The anti-pSTAT3^Tyr705^ (1 : 500, #9145; Cell Signaling, Danvers, MA, USA) was incubated with tissue sections at 4°C overnight. Subsequently, the slices were incubated with a secondary antibody and color-developed using DAB according to the manufacturer's recommendations.

### 2.5. RNA Interference

Two different PTPRK small interfering RNA (siRNA) fragments and nonspecific control siRNA (siRNA NC) were designed and synthesized by Sigma-Aldrich. The PTPRK sense sequences are as follows: siRNA#1, 5′-CGAUUAUCCACUGCCUAAAdTdT-3′ and siRNA#2, 5′-GUGAUGUGAUCAACCGGAUdTdT-3′. The siRNAs/siRNA NC was transfected into the cells to knock down the PTPRK by Lipofectamine 2000 (Invitrogen, USA). 48 h after transfection, the cells were collected for further assays.

### 2.6. Cell Proliferation Assays

Cell proliferation was determined by CCK-8 kit (Beyotime, China) [13]. Briefly, 3 × 10^3^ of the transfected H1299 and A549 cells were seeded into each well of a 96-well plate and cultured for 6 h-72 h. At the end of different experimental periods (6, 24, 48, and 72 h), 10 *μ*L CCK-8 solution was added to each well for 2 h incubation at 37°C. Cell viability was determined by reading OD (optical density) at a wavelength of 450 nm using a microplate reader.

### 2.7. Wound Healing Assay

The migration ability of H1299 and A549 cells was performed in a classical wound healing assay [13]. Briefly, cells grown in six-well tissue culture dishes with 80% confluence were manually scratched with a 10 *μ*L tip. The scrapes with uniform width were created through the confluent monolayer. The cells were replaced in the fresh culture medium and incubated at 37°C. Images of the initial wound and the movement of cells into the scratched area were observed and photographed by using an inverted microscope (Leica DMIL, Germany) at 0 and 20 h.

### 2.8. Transwell Invasion Assay

Cell invasion ability was detected using the Cell Invasion Assay Kit (no. ECM550, Millipore, USA) [13]. Briefly, H1299 and A549 cells were harvested 24 h after transfection and seeded at a density of 1 × 10^5^ cells into the upper chamber. Subsequently, 500 *μ*L of RPMI-1640 medium with 10% fetal bovine serum was added to the lower chamber. Following a 24 h incubation at 37°C, the cells remaining on the upper surface of the membranes were carefully wiped out. Invasive cells on the lower surface of the membrane were stained by dipping inserts in the staining solution for 20 minutes. Five random fields were counted per chamber using an inverted microscope (Leica DMIL, Germany), and each experiment was repeated in triplicate.

### 2.9. Western Blotting Analysis

The cells were lysed and isolated in NETN lysis buffer (20 mM Tris-HCl (pH 8.0), 100 mM NaCl, 1 mM EDTA, and 0.5% Nonidet P-40) supplemented with 1 mM PMSF (Beyotime, Haimen, China) [13]. Protein concentration was assessed using a Quick Start Bradford protein assay kit (Bio-Rad). Equal amount of total protein was boiled and separated by 10% SDS-PAGE and transferred onto the PVDF membrane. The blotted membrane incubated with diluted specific antibodies at 4°C overnight and goat anti-rabbit secondary antibody (1 : 10000, Jackson ImmunoResearch, USA) subsequently. The target bands were detected by ECL chromogenic substrate. STAT3 (#12640) and pSTAT3 (#9145) antibodies were purchased from Cell Signaling Technology Inc. PTPRK (ab185370) and *β*-actin (ab8227) antibodies were purchased from Abcam. Western blot quantification was performed using ImageJ software.

### 2.10. Statistical Analysis

Statistics 18.0 software (SPSS Inc., Somers, NY, USA) was used to perform the statistical analyses in this study. Mann-Whitney test was used to compare the mean value of mRNA levels between the two groups. One-way ANOVA with a Bonferroni posttest to analyze the difference between the 16HBE and seven lung cancer cells. It was considered significant when the *p* value was less than 0.05.

## 3. Results

### 3.1. PTPRK Is Frequently Underexpressed in NSCLC with Lymph Node (LN) Metastasis

**Figure 1:**
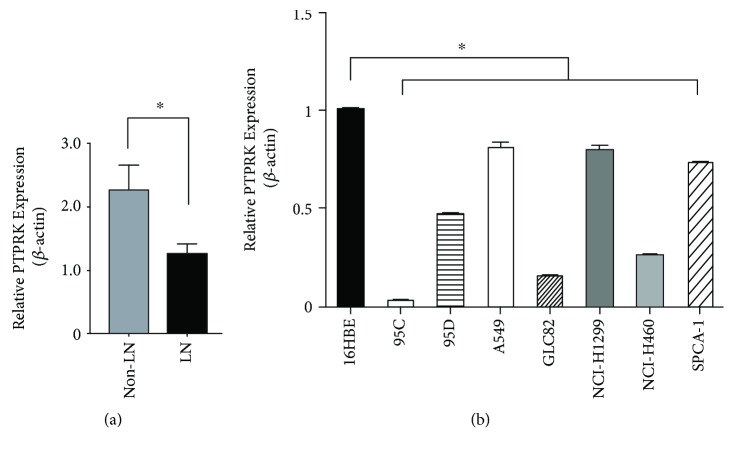
PTPRK is frequently underexpressed in NSCLC with lymph node (LN) metastasis. (a) PTPRK mRNA expression was quantified by qRT-PCR in 30 lung tumors with non-lymph node metastasis and 16 tumors with lymph node metastasis. (b) qRT-PCR analysis of PTPRK expression levels in one normal human bronchial epithelial cell (16HBE) and seven NSCLC cell lines, and expression levels were all normalized to 16HBE.

To establish the association between PTPRK expression and tumor metastasis, the PTPRK mRNA expression level was measured by qRT-PCR analysis in 30 lung tumors with non-lymph node metastasis and 16 tumors with lymph node metastasis. As shown in Figure 1(a), we found that mRNA levels of PTPRK were significantly lower in the lymph node metastasis group compared to the non-lymph node metastasis group (*p* = 0.045). Similarly, the PTPRK levels in seven NSCLC cell lines (95C, 95D, A549, GLC82, NCI-H1299, NCI-H460, and SPCA-1) were significantly lower than those in the normal lung cell line (16HBE) (*p* < 0.001, Figure 1(b)).

### 3.2. PTPRK Knockdown Abolishes Its Oncosuppressive Function in H1299 Cells

**Figure 2:**
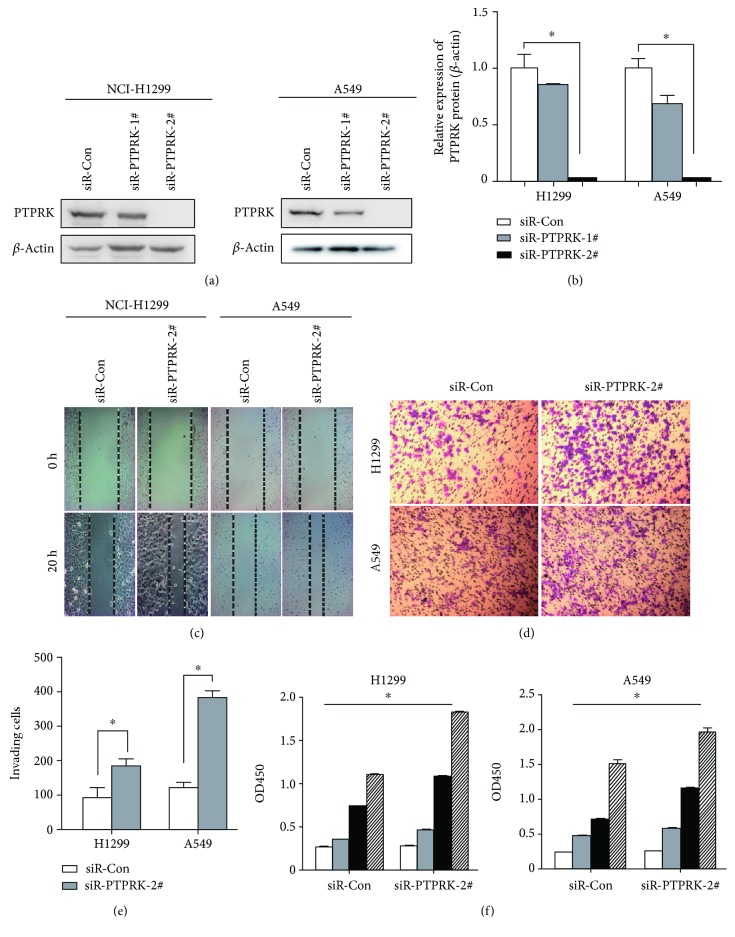
PTPRK knockdown promotes the cell proliferation, migration, and invasion ability in H1299 and A549 cells. (a) Western blotting analysis protein H1299 and A549 cells transfected two chemically synthesized siRNAs. (b) Quantitative analysis of PTPRK protein levels was calibrated with beta-actin levels of each sample from (a). (c) Representative micrographs of wound healing assay of the H1299 and A549 cells transfected with PTPRK siRNA#2 or NC. Wound closures were photographed at 0 h and 20 h after wounding. (d) Representative micrographs of Transwell invasion assay of the H1299 and A549 cells transfected with PTPRK siRNA#2 or NC. (e) Quantification of indicated invading cells in five random fields analyzed by the Transwell assays. Values represent the mean ± SD from three independent measurements. (f) Cell proliferation assays. H1299 and A549 cells were transfected with PTPRK siRNA#2 or NC. Cells were counted by a CCK-8 kit after 6 h, 24 h, 48 h, and 72 h. Values represent the mean ± SD from three independent measurements.

To determine whether PTPRK contributes to the metastatic abilities of lung cells, we used two chemically synthesized siRNAs to knock down endogenous PTPRK in H1299 and A549 cells. After 48 h posttransfection, PTPRK protein expression levels were effectively 75% knocked down by siR-PTPRK-2# as determined by western blot analysis (Figures 2(a) and 2(b)). The results showed that PTPRK knockdown strongly promoted the migratory ability with a closer gap compared to the control (Figure 2(c)). Similarly, we also observed an increased invading capacity after siRNA-mediated silencing of PTPRK (Figures 2(d) and 2(e)). Additionally, silencing PTPRK in H1299 and A549 cells significantly promoted cell proliferation (Figure 2(f)). Collectively, our results validated the PTPRK-mediated tumor suppressor functions by inhibiting proliferation and metastasis of lung cancer cells.

### 3.3. PTPRK Downregulation Contributes to STAT3 Activation and Is Associated with Poor Prognosis of NSCLC

**Figure 3:**
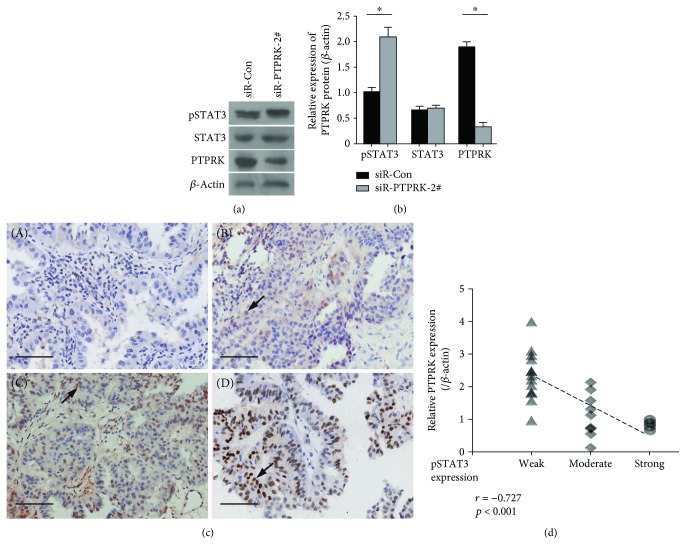
PTPRK downregulation contributes to STAT3 activation and is associated with poor prognosis of NSCLC. (a) The protein expression level of phopho-STAT3^Tyr705^, STAT3, and PTPRK was measured by western blotting in H1299 cells transfected with NC and PTPRK siRNA#2. (b) Densitometric quantifications of phopho-STAT3^Tyr705^, STAT3, and PTPRK protein levels in H1299 cells transfected with NC and PTPRK siRNA#2 according to (a). (c) Representative micrographs of immunohistochemical staining of the phopho-STAT3^Tyr705^ protein (brown nuclear staining) in 26 NSCLC tissues. (A) negative control; (B) weak positive (+) expression, weak staining pattern; (C) positive expression (++), medium staining pattern; (D) strong positive expression (+++), strong staining pattern. (d) Correlation between phopho-STAT3^Tyr705^ and PTPRK expression was analyzed. Expression of PTPRK in 26 clinical tissue samples was measured by real-time PCR.

STAT3 is persistently activated in approximately 50% of NSCLC primary tumors and lung cancer-derived cell lines. Recent studies showed that the PTPRK gene contains a STAT3-specifying motif, which negatively regulates STAT3 activation in NKTCL [9]. Therefore, we explored whether the downregulation of PTPRK leads to STAT3 activation in lung cancers. Indeed, PTPRK depletion significantly increased the levels of phospho-STAT3^Tyr705^ in H1299 cells (Figures 3(a) and 3(b)). Importantly, the expression of phospho-STAT3^Tyr705^ was significantly inversely correlated with the mRNA levels of PTPRK in 26 NSCLC tissues (*r* = −0.727, *p* < 0.001, Figures 3(c) and 3(d)), and high expression of phospho-STAT3^Tyr705^ was positive correlated with lymph node metastasis of patients with NSCLC (*p* = 0.041) (Table 1).

## 4. Discussion

Protein tyrosine phosphatases (PTPs), the homeostatic counterpart of PTKs, are critical regulators that control cellular homeostasis. PTPRK, which is located in the frequently deleted gene region 6q22.2-22.3 of various tumors, was identified as a candidate tumor suppressor gene in cancer research [14, 15]. Several studies had reported lower levels of PTPRK transcripts in breast cancer, colon cancer, and rhabdoid tumors [5, 7, 16]. In the current study, our results detected a lower PTPRK expression in NSCLC with lymph metastasis tissues and cell lines (Figure 1). Our recent study showed that PTPRK is a direct target of miR-1260b in NSCLC cells, and the downregulation of PTPRK is associated with poor prognosis of patients with NSCLC [13]. Previous data from a breast cancer research demonstrated decreased PTPRK levels in the primary breast tumors [7]. The same tendency for survival time was found in CNSL patients [10].

After reducing PTPRK expression *in vitro*, we observed the strongly promoted capabilities of cell proliferation, invasiveness, and migration, as expected for a tumor suppressor in lung cancer (Figure 2). It hinted towards a negative role of PTPRK in lung cancer invasion and metastasis, which is consistent with its function in other cancers. Specifically, the downregulation of PTPRK in breast cancer cell lines was associated with increased cell proliferation, adhesion, and invasion [7].

Signal transducer and activator of transcription 3 (STAT3), a vital prooncogenic transcription factor in multiple cancers [17–19], is constitutively activated in approximately 50% of NSCLC primary tumors and lung cancer-derived cell lines and may be one of the most important oncogenic drivers in NSCLC [11, 20]. STAT3 phosphorylation at Tyr705 by diverse upstream kinases, including cytokine receptors and tyrosine kinases, is a key step for STAT3 activation, which induces transcription of a wide array of genes that play critical roles in lung cancer pathogenesis [15, 21]. Here, we found that the phospho-STAT3^Tyr705^ levels of NSCLC tissues were positively correlated with lymph node metastasis and significantly inversely correlated with the expression of PTPRK silencing.

PTPRK increased the expression of phospho-STAT3^Tyr705^ in NSCLC cells (Figure 3). These results indicate that the loss of PTPRK expression likely contributes to tumorigenesis by activating the STAT3 oncoprotein in NSCLC. Normally, activated STAT3 protein can be dephosphorylated promptly by protein phosphatases [22]. However, STAT3 is constitutively active in most lung cancers. Therefore, the decreasing activity of PTPRK may be partly accountable for the constitutive activation of STAT3 in lung cancers.

In conclusion, we demonstrated that the PTPRK protein serves a significant role in the lung tumor proliferation and metastasis via its ability to inhibit STAT3 activity in NSCLC. Our findings show PTPRK as an important tumor suppressor and a potential target gene for diagnosis and therapies in NSCLC.

## Figures and Tables

**Table 1:** Association of pSTAT3 expression with clinicopathological parameters in 26 NSCLC specimens.

Parameters	Case	pSTAT3 expression		*p* value
		Weak	Moderate/strong	
Age (years)				
≤65	20	8	12	1
>65	5	3	2	
Tumor size (cm)				
≤3	8	5	3	0.401
>3	18	7	11	
Grade				
I–II	18	8	10	1
III-IV	8	4	4	
LN metastasis				
Negative	10	8	2	**0.041** ^∗^
Positive	16	5	11	

## Data Availability

The data that support the findings of this study are available from the corresponding author upon reasonable request.

## Abbreviations

PTPRK: Protein tyrosine phosphatase, receptor type K
STAT3: Signal transducer and activator of transcription 3
NSCLC: Non-small-cell lung cancer.
